# Corticofugal projections induce long-lasting effects on somatosensory responses in the trigeminal complex of the rat

**DOI:** 10.3389/fnsys.2014.00100

**Published:** 2014-05-22

**Authors:** Eduardo Malmierca, Irene Chaves-Coira, Margarita Rodrigo-Angulo, Angel Nuñez

**Affiliations:** Departamento de Anatomía, Histología y Neurociencia, Facultad de Medicina, Universidad Autónoma de MadridMadrid, Spain

**Keywords:** principal trigeminal nucleus, caudal spinal trigeminal nucleus, somatosensory cortex, insular cortex, nociception, corticofugal projection

## Abstract

The sensory information flow at subcortical relay stations is controlled by the action of topographic connections from the neocortex. To determinate the functional properties of the somatosensory corticofugal projections to the principal (Pr5) and caudal spinal (Sp5C) trigeminal nuclei, we performed unitary recordings in anesthetized rats. To examine the effect of these cortical projections we used tactile stimulation of the whisker and electrical stimulation of somatosensory cortices. Corticofugal anatomical projections to Pr5 and Sp5C nuclei were detected by using retrograde fluorescent tracers. Neurons projecting exclusively to Pr5 were located in the cingulate cortex while neurons projecting to both Sp5C and Pr5 nuclei were located in the somatosensory and insular cortices (>75% of neurons). Physiological results indicated that primary somatosensory cortex produced a short-lasting facilitating or inhibiting effects (<5 min) of tactile responses in Pr5 nucleus through activation of NMDA glutamatergic or GABA_A_ receptors since effects were blocked by iontophoretically application of APV and bicuculline, respectively. In contrast, stimulation of secondary somatosensory cortex did not affect most of the Pr5 neurons; however both cortices inhibited the nociceptive responses in the Sp5C nucleus through activation of glycinergic or GABA_A_ receptors because effects were blocked by iontophoretically application of strychnine and bicuculline, respectively. These and anatomical results demonstrated that the somatosensory cortices projects to Pr5 nucleus to modulate tactile responses by excitatory and inhibitory actions, while projections to the Sp5C nucleus control nociceptive sensory transmission by only inhibitory effects. Thus, somatosensory cortices may modulate innocuous and noxious inputs simultaneously, contributing to the perception of specifically tactile or painful sensations.

## Introduction

The flow of sensory information at subcortical relay stations is controlled by the action of precise topographic connections from the neocortex. The trigeminal nuclei are the first relay stations in the ascending somatosensory system, which receive information from the ipsilateral face (Erzurumlu et al., 2010). The principal sensory trigeminal (Pr5) nucleus conveys whisker-specific non-nociceptive tactile sensory information to the ventro-posteromedial nucleus of the thalamus and subsequently to the primary somatosensory (S1) cortex as well as the secondary somatosensory (S2) cortex in the rat. The caudal division of the trigeminal spinal (Sp5C) nucleus has been viewed as an essential brainstem site for relaying facial pain information to higher levels of the central nervous system (for review see Bereiter et al., 2000; Sessle, 2006). *In fact, hyperalgesia evoked by infraorbital nerve constriction induce an increase of excitability of Sp5C neurons* (Okubo et al., 2013). Both nuclei have a complete whisker representational maps, however, sensory processing may be different depending on their sensory afferents (Timofeeva et al., 2005; Furuta et al., 2010; Mosconi et al., 2010).

The S1 and S2 cortices have descending projections to subcortical relay neurons that modulate the ascending sensory information (Wise et al., 1979; Rustioni and Hayes, 1981; Malmierca and Nuñez, 1998; Canedo and Aguilar, 2000; Martinez-Lorenzana et al., 2001; Aguilar et al., 2003; Malmierca and Nuñez, 2004; Noseda et al., 2010; Tomita et al., 2012). However, the functional significance of S1 and S2 corticofugal projections to the trigeminal neurons are poorly understood. Anatomical results indicate that the orofacial S1 and S2 cortices selectively project to trigeminal nuclei, which may accurately modulate orofacial somatosensory transmission to higher brain centers (Haque et al., 2012). Nevertheless, the precise projection pattern from the somatosensory cortices to the trigeminal nuclei is not well established.

In rats, S2 cortical neurons may play a secondary role in discriminative somatosensory functions since these neurons have wider receptive fields (RFs) than S1 neurons (Carvell and Simons, 1986; Alloway et al., 2000). Thus, the main function of S2 neurons could be integrative somatosensory processing since the S2 cortex contains many multisensorially responsive neurons (Mima et al., 1998; Romo et al., 2002; Inui et al., 2003; Torquati et al., 2003; Brett-Green et al., 2004; Menzel and Barth, 2005).

Given that the somatosensory cortex conveys non-nociceptive and nociceptive sensory information while nociceptive sensory information is mainly analyzed in the Sp5C nucleus, the goal of the present work is to determine both the functional properties of the somatosensory corticofugal projections to the trigeminal nuclei in the rat as well as whether or not cortical neurons might be segregated into different populations according to the origin of their pre-thalamic afferences (Pr5 or Sp5C). We have studied whether or not a single somatosensory cortical neuron projects to both the Pr5 and Sp5C nuclei and the electrophysiological effect this may have in these pre-thalamic nuclei.

## Materials and methods

### General experimental procedures

Electrophysiological experiments were performed on 49 adult Sprague-Dawley rats of either sexes weighing 250–300 g (from Iffa-Credo, France). Anatomical experiments were performed in 11 adult Sprague-Dawley rats (RE-1 to R-11) weighing 230–290 g. Animals were housed under standard colony conditions and food and water were supplied *ad libitum*. All animal procedures were approved by the Ethical Committee of the Autonomous University of Madrid, in accordance with European Community Council Directive 2010/63/UE. Efforts were made to minimize animal suffering as well as to reduce the number of animals used.

### Anatomical experiments

The anatomical pathways linking cortical areas with the Pr5 and Sp5C trigeminal nuclei were studied by injecting or depositing the neuroanatomical fluorescent retrograde tracers Fluoro-Gold (FlGo; injection; Fluorochromes, Llc. Denver, USA) and Fast Blue (FB; deposit; Polysciences, Inc. Warrington, PA) respectively in the Pr5 and Sp5C nuclei in rats.

Animals (*n* = 11) were anesthetized with an intraperitoneal injection of a mixture of ketamine (70 mg/Kg) and xylazine (5 mg/Kg) with supplementary doses when necessary (35 mg/Kg and 2.5 mg/Kg respectively) and placed in the stereotaxic frame. After appropriate craniotomy, 60 nl of a 4% saline dilution of FlGo was injected in the Pr5 nucleus by means of a 1 µl Hamilton syringe at stereotaxic coordinates: antero-posterior −9.5 from Bregma, lateral 2.8 and vertical 8.8 according to Paxinos Atlas (Paxinos and Watson, 2007). After surgically separating the nucal musculature we accessed the cisterna magna by opening the dura mater to expose the Sp5C nucleus, whose superficial location in the medulla is easy to recognize. Deposits of 2 mm^2^ pieces of absorbable gelatin “Spongostan” embedded in a 1% saline solution of FB were placed on the Sp5C nucleus for 15 min. Once muscles and wounds were sutured animals were treated with Metacam 1 mg/Kg and Dalsy (15 mg/Kg diluted in drinking water). After a survival period of 1 week animals were anesthetized with an overdose of the same anesthesia and perfused transcardially with 4% paraformaldehyde in 0.1 M phosphate buffer at pH 7.3 followed by increasing concentrations of sucrose solutions (5%, 10%, 20%) in the same buffer. Brains were stored in 30% sucrose for at least 5 days for tissue cryopreservation and frozen sectioned on the coronal plane at 40 µm; sections were collected in three consecutive ordered series devoted to Nissl staining, cytochrome-oxidase staining, and fluorescent visualization. Sections containing the cerebral cortex of the fluorescent visualization series were studied under a Nikon Axioskop fluorescent microscope. Sections for cytochrome oxidase staining were incubated for 3 h in the complex solution (Phosphate Buffer, DAB (Sigma), Cytochrome C (Sigma C-7752) and sucrose (Microbiologie) before being studied under an optical/fluorescent microscope, so both cytochrome and fluorochrome labeled cells could be observed. Series processed for Nissl staining were used for delimiting structures.

For a quantitative study, selected sections were also analyzed under a confocal microscope (Leica TCS SP5) using the Tile Scan tool of LAS AF software; samples were analyzed under both lin 405 mm UV and lin Ar 488 mm using a 10x objective. Images are a stack of sections in maximal projection, but neurons were counted in each individual layer.

### Electrophysiological recordings

Data were obtained from urethane-anesthetized (1.6 g/Kg i.p.) Sprague-Dawley rats. Supplemental doses of urethane (0.5 g/Kg i.p.) were given to maintain areflexia. Animals were placed in a Kopf stereotaxic device in which surgical procedures and recordings were performed. The body temperature was maintained at 37°C; the end-tidal CO_2_ and heart rate were monitorized.

Single unit recordings were performed by using tungsten microelectrodes (2–5 MΩ; World Precission Instruments) in the Pr5 nucleus (antero-posterior −9.5 from Bregma, lateral 2.8 and vertical 8.8 mm according to Paxinos and Watson, 2007) or the Sp5C nuclei (antero-posterior −14.3 from Bregma, lateral 3 and vertical 0.5–2 mm from the surface; the recording electrode was placed at a 60° angle to the surface of the nucleus after opening the cisterna magna). Unit firing was filtered (0.3–3 KHz), amplified via an AC preamplifier (P15; Grass Instruments) and fed into a personal computer (sample rate: 10 KHz) with the stimuli events for off-line analysis using Spike 2 software (Cambridge Electronic Design, Cambridge, UK).

To record the electroencephalogram (EEG) a macroelectrode (120 µm diameter bluntly cut insulated nichrome wire) was lowered 0.5 mm from the cortical surface into the somatosensory cortex. The EEG was filtered between 0.3 and 30 Hz and sampled at 100 Hz. EEG was used to test the level of anesthesia and to correlate the trigeminal neuronal firing with the EEG activity.

### Sensory and cortical stimulation

Whisker deflection was performed with a brief, electronically gated, air puff (1–2 Kg/cm^2^, 20 ms duration; Picospritzer) delivered at 0.5 Hz through a 1 mm inner diameter polyethylene tube. To avoid complex responses due to deflections of multiple vibrissae, these were trimmed to 5 mm long, allowing reproducible responses to be evoked. RFs were defined by the limits at which stimuli elicited spike responses.

To place the stimulating electrode, the bone over the contralateral S1 or S2 cortex was removed. Prior to placing the stimulating electrode at the S1 or S2 cortex, a tungsten microelectrode for multiunit recording (0.5–1 MΩ; World Precision Instruments) was aimed at the cortex (A: −1 to −3 mm, L: 3–6 mm, 0.5–1 mm deep) to locate a site with a vigorous multiunit response to tactile stimulation of the whisker. After detecting the RF in the S1 or S2 cortex, a bipolar stimulating electrode (120 mm diameter blunt cut stainless steel wire) was aimed at the same site as the recording electrode (1 mm depth; cortical layer 5). Single pulses (0.3 ms duration; 50–200 µA current intensity) or barrages of pulses (3 trains of stimuli at 50 Hz during 500 ms repeated each 2 s) were applied to the S1 or S2 cortex through bipolar electrodes (100 µm diameter blunt cut insulated stainless steel wires; World Precision Instruments) by a Grass S88 stimulator coupled to a photoelectric stimulus isolation unit. The experimental protocol consisted in a period of control tactile stimulation (30 air jets delivered during 60 s to the principal whisker) followed by 5 min of continuous tactile stimulation after the cortical stimulation barrages.

A bipolar stimulating electrode was positioned at stereotaxic coordinates in the ventroposterior medial nucleus of the thalamus (VPM; antero-posterior −4.0 from Bregma, lateral 3.0 and vertical 6.5 mm) in order to identify thalamic projecting neurons. Pulses of electrical current (100–500 µA, 0.3 ms) were applied through the stimulating electrode. Antidromically-activated neurons were identified by a constant response latency to VPM stimulation and the ability to follow high frequency stimulus train (>100 Hz).

In some cases small electrolytic lesions (10 µA, 15 s) were made at the end of the recording session to identify the stimulating and recording electrodes in Nissl stained brain sections.

### Pain induction

The activity of Pr5 and Sp5C neurons was evaluated in control conditions and after unilateral application of capsaicin cream (1.6%) on the vibrissa pad, affecting one or two whiskers. The cream did not impede the movement of the whiskers. Topical application of capsaicin cream induces excitation of nociceptors and, consequently, pain lasting several hours (Yoshimura and Yonehara, 2001; Martin and Avendaño, 2009; Martin et al., 2010).

### Drug application

The selective GABA_A_ receptor antagonist bicuculline (10 mM in 0.9% NaCl, pH 3, Sigma, St. Louis, MO, USA) or the glycinergic receptor antagonist strychnine hydrochloride (10 mM in 0.9% NaCl, pH 3, Sigma) were applied iontophoretically with a multibarrel micropipette (20 µm tip diameter). The NMDA receptor antagonist D-2-amino-5-phosponovaleric acid (APV; 50 mM, pH 8) was also applied iontophoretically. A barrel was filled with 1 M NaCl for extracellular recording and a second micropipette was filled with APV, strychnine or bicuculline. The remaining micropipette, filled with 1 M NaCl solution, balanced the currents. Each barrel of the three-barreled pipette was connected via a silver wire to a channel of a microiontophoresis current generator (WPI current generator). The current generator controlled retention and ejection currents for the drug-filled micropipette. Drugs were ejected with negative (for APV) or positive (for bicuculline and strychnine) current, using a single 10–30 s pulse of up to 200 nA. Retaining currents of 10–20 nA were used to delay drug leakage from the barrel. Since the current pulse used to inject drugs had a small intensity, they only affected the close area around the multibarrel micropipette.

### Data analysis

Recordings were accepted for statistical analysis when the spike amplitude fluctuations were lower than 10% throughout the experiment. Peristimulus-time histograms (PSTHs; 1 ms bins) were calculated from 30 stimuli using the Spike 2 software. The mean tactile response was measured from the PSTH as the number of spikes evoked at 0–50 ms after stimulus onset divided by the number of stimuli. We considered that neurons responded to tactile stimulation when the cell discharged at least one spike every two stimuli. The autocorrelogram time histogram (ACH) was also calculated to reveal oscillatory activity. A paired *t*-test was used for comparisons. All data are shown as mean ± standard error.

## Results

### Corticofugal projection to trigeminal complex

**Figure 1:**
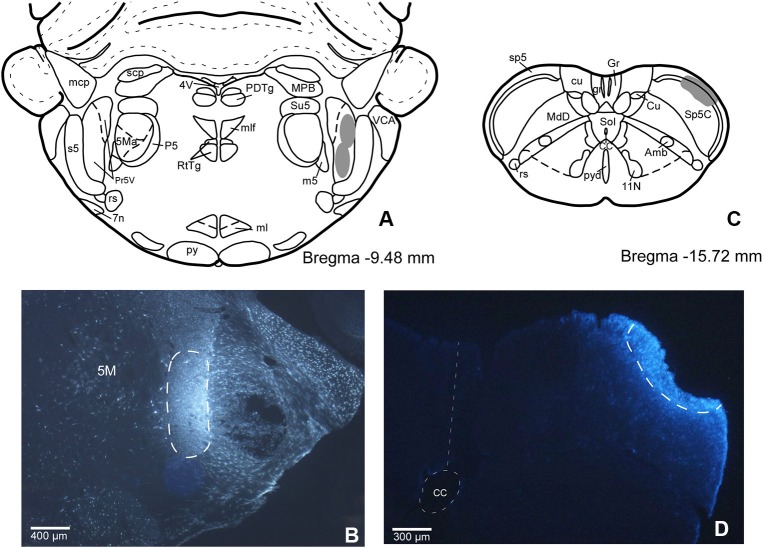
**(A)** Schematic drawing of Bregma −9.50 mm Paxinos and Watson Atlas. The gray area represents the summarized location in Pr5 of the FlGo injections in all cases. **(B)** Microphotograph of a coronal section in animal RE11 showing the injection side in Pr5 (limited by dashed line). **(C)** Schematic drawing of Bregma −15.7 mm showing the limits of the FB injection in Sp5C represented by gray area. **(D)** Microphotograph of a coronal section showing the injection site in Sp5C (limited by dashed line) in animal RE8. Calibration toolbar: B: 400 µm, D: 300 µm. Abbreviations: 11N: accessory nerve nucleus, 4V: 4th ventricle, 5M: motor trigeminal nucleus, 5Ma: motor trigeminal nucleus, masseter part , 7n: facial nerve, Amb: ambiguous nucleus, CC: central canal, Cu: cuneate nucleus, cu: cuneatus fasciculus, gr: gracile fasciculus, Gr: gracile nucleus, ml: medial lemniscus, mlf: medial longitudinal fasciculus, mcp: middle cerebellar peduncle, P5: peritrigeminal zone, PDTg: posterodorsal tegmental nucleus, Pr5V: principal sensory trigeminal nucleus, py: pyramidal tract, pyd: pyramidal decussation, rs: rubrospinal tract, RtTg: reticulotegmental nucleus of the pons, s5: sensory root of the trigeminal nerve, scp: superior cerebellar peduncle (brachium conjunctivum), Sol: nucleus of the solitary tract, sp5: spinal trigeminal tract, Su5: supratrigeminal nucleus, m5: motor root of the trigeminal nerve, MdD: medullary reticular nucleus, dorsal part, MPB: medial parabrachial nucleus, VCA: ventral cochlear nucleus, anterior part.

In order to establish if there are individual or shared corticofugal projections from S1 and S2 to Pr5 and Sp5C trigeminal nuclei, we have injected two different fluorescent retrograde tracers in both trigeminal nuclei. This anatomical study was performed on 11 rats. The locations of the FlGo injection site in Pr5, as well as the FB deposit in Sp5C were confirmed on the sections reserved for fluorescence study of those trigeminal nuclei. Both injections and deposits were confined to the desired site without signals of diffusion in any case (Figure 1).

**Figure 2:**
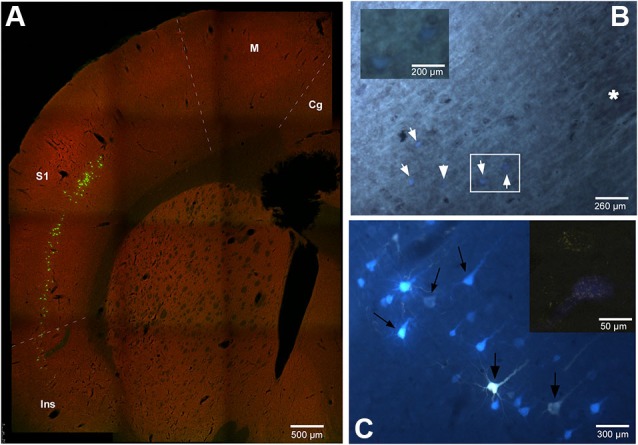
**(A)** Confocal microscope microphotograph of a coronal section of the brain in animal RE9 at Bregma 2.16 mm stereotaxic coordinates, showing double labeled neurons in S1 and Ins cortices. **(B)** Microphotograph showing the barrel zone of S1 cortex in animal RE8 where the fluorescence was combined with cytochrome oxidase technique. Asterisk corresponds to the barrel zone, white arrows points the retrograde labeled neurons. Cytochrome oxidase labeled neurons located in the white square, are magnificated in the inset. **(C)** Microphotograph of S1 cortex in animal RE9 showing the FlGo and FB labeled neurons. Black arrow shows the double labeled neurons. Inset: Confocal microscope detail of a single FlGo labeled neuron (up) and double labeled neuron (down). Calibration toolbar: A: 500 µm, B: 260 µm, inset 200 µm C: 300 µm, inset 50 µm. Abbreviations: Cg: cingulate cortex, Ins: insular cortex, M: motor cortex, S1: primary somatosensory cortex.

**Figure 3:**
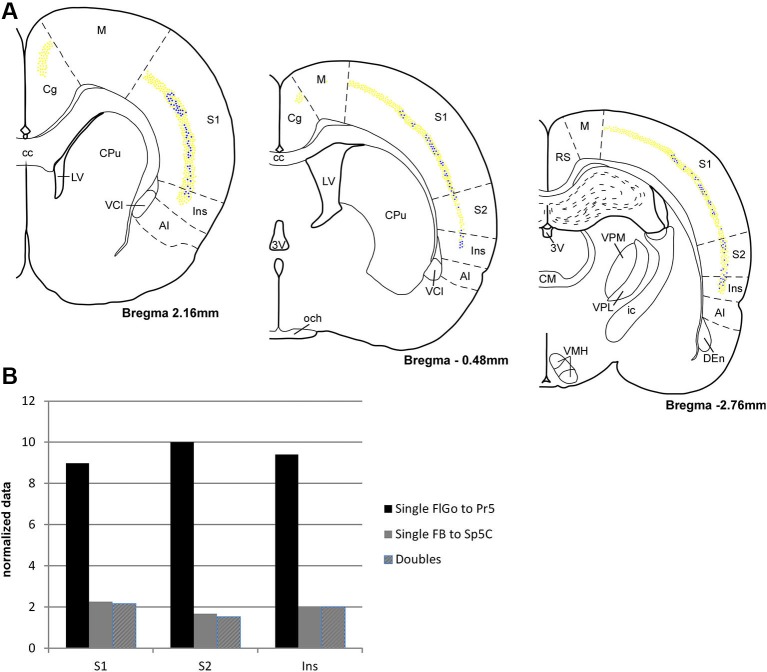
**(A)** Schematic drawings of coronal hemi-sections from rostral to caudal levels through rat brain showing the distribution FLGo (light dots) and FB (dark dots) labeled neurons in different cortical areas. **(B)** Graphic representation of percentages of single FlGo, single FB and double-labeled neurons in different cortical areas. Abbreviations: 3V: 3rd ventricle, AI: agranular insular area, CC: central canal, Den: dorsal endopiriform nucleus, Cg: cingulate cortex, CM: central medial thalamic nucleus, CPu: caudate putamen (striatum), ic: internal capsule, Ins: insular cortex, LV: lateral ventricle, M: motor cortex, och: optic chiasm, RS: retrosplenial cortex, S1: primary somatosensory cortex , S2: secondary somatosensory cortex, VCl: ventral part of claustrum, VMH: ventromedial hypothalamic nucleus, VPL: ventral posterolateral thalamic ucleus, VPM: ventral posteromedial thalamic nucleus.

The location and relative amount of retrogradely labeled neurons by one (single labeled neurons) or both (double labeled neurons) tracers used in the experiment have been studied in the cerebral cortex of the animals. In every case the retrogradely labeled neurons were located in layer V, mainly in the cingulate, S1, S2 and insular (Ins) cortices (Figure 2). Figure 2C shows case RE-9 in which pyramidal cells of the S1 cortex were labeled by both retrograde tracers, FlGo and FB, after injections in the Pr5 and Sp5C nuclei respectively. Remarkably, groups of single- and double-labeled neurons were confined to the barrel zones all over S1 cortex, as confirmed by the study of the cytochrome-oxidase reaction combined with fluorescence visualization (Figure 2B). Single FlGo-labeled neurons (projecting exclusively to Pr5 nucleus) were only located in the cingulate cortex (quantitative data not shown) while double labeled neurons (projecting to the Sp5C and Pr5 nuclei) were located in the S1, S2 and Ins cortices (Figures 2A, C and 3). Occasionally (<3%), single FB labeled neurons were observed in these cortices (S1, S2 and Ins).

For the quantitative analysis, single FlGo and FB labeled neurons were counted separately; first double-labeled neurons were counted in all sections and then result were contrasted to the superposition of single labeled neurons in all the layers of the confocal stack. The mean percentages of labeled neurons have been reported as the total FlGo, FB and double-labeled neurons in each cortical area (Figure 3). The mean percentages of single FlGo labeled neurons were considerably higher (97.4%) than the single FB labeled neurons (2.6%). Data shown in the graph in Figure 3B have been normalized.

Single FlGo labeled neurons were present in all the rostro-caudal levels of the cingulate cortex (Figure 3); however since we were not able to find single FB or double-labeled neurons in this cortex, the quantitative data are not shown. A considerable amount of homogenously distributed single FlGo labeled neurons (77.6%) was observed in S1 cortex, while the distribution of double-labeled neurons (22.4%) changed progressively, being found dorsally at rostral levels and more ventrally at caudal levels (Figure 3). In S2 cortex 87.7% of neurons were single FlGo labeled neurons and 12.3% were double-labeled neurons; however the distribution of double-labeled neurons was not as homogeneous as in S1 cortex, since the number of these neurons decreased progressively from the region adjacent to the S1 cortex to the region close to the Ins cortex (Figure 3). Finally, the Ins cortex was 76.6% single FlGo labeled neurons compared to the 23.4% of homogeneously distributed double-labeled neurons. All percentages shown in Figure 3 in the S1, S2 and Ins cortices are normalized.

The percentage of single FB labeled neurons (projecting to Sp5C nucleus) was similar in the S1, S2 and Ins cortices (<2%). This means that most of the cortical neurons that project to the Sp5C nucleus are double-labeled cells that also project to the Pr5 nucleus.

### Neuronal population and tactile responses of Pr5 and Sp5C neurons

**Figure 4:**
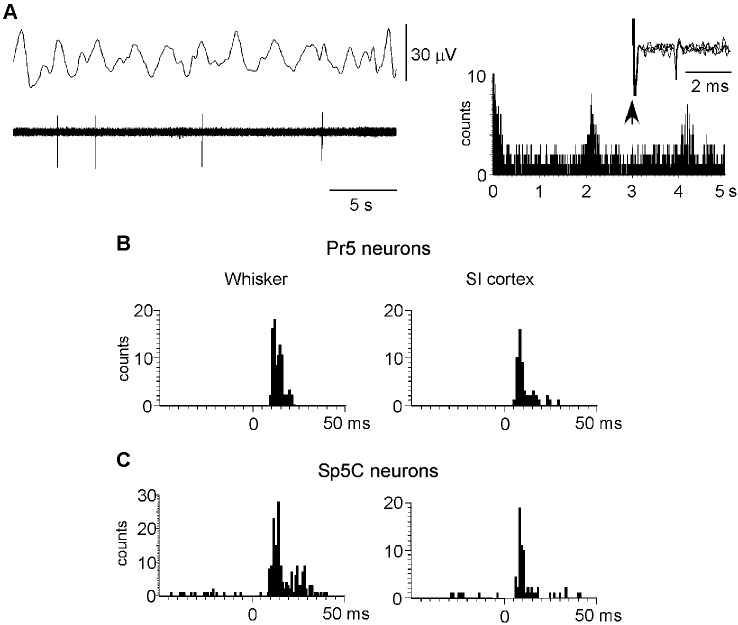
**Neuronal characteristics of Pr5 and Sp5C neurons. (A)** Raw data of a representative Pr5 neuron. Neuron shows a low firing rate but spikes tended to occur in the positive wave of the EEG. The ACH on the right shows that spikes occurred with an interval of 2.1 s. In this case, the Pr5 neuron was antidromically identified as thalamic- projecting neuron because VPM electrical stimulation induced antidromic spikes (three traces are superimposed). **(B)** PSTHs of responses evoked by whisker stimulation or S1 cortex stimulation (left and right histograms, respectively) in a representative Pr5 neuron. **(C)** same PSTHs in a Sp5C neuron. PSTHs are the sum of 30 trials.

To study the cortical influence on Pr5 and Sp5C neuronal activity, unit recordings were obtained from Pr5 (*n* = 104), sampled throughout the entire dorsoventral extension of the nucleus, or from lamina III–V of the Sp5C nucleus (at 800–1200 mm deep from the surface *n* = 70). The Pr5 and Sp5C neurons were silent under spontaneous conditions or displayed a low firing rate (<1 spikes/s) that followed slow EEG activity. Note that spikes tended to occur in the positive wave of the EEG (Figure 4A). The mean firing rate was 0.6 ± 0.35 spikes/s and 0.4 ± 0.2 spikes/s for Pr5 neurons and Sp5C neurons, respectively. The ACH of trigeminal neurons showed peaks at intervals of 2–4 s that corresponded with the frequency of the slow EEG activity. Thus, results indicated that this slow oscillation of the trigeminal spontaneous activity was imposed by the slow cortical activity described previously (Steriade et al., 1993; Figure 4A, right histogram).

To determine if recorded neurons were thalamic-projecting neurons electrical stimuli were applied to the VPM nucleus (50–200 µA; 0.3 ms duration). Eleven out of 14 Pr5 neurons (78%) and 11 of 18 Sp5C neurons (61%) could be activated antidromically. Antidromic spikes were identified by the fixed latency (2.4 ± 0.6 ms and 3.1 ± 0.5 ms for Pr5 and Sp5C neurons, respectively) and the ability to follow stimuli above 100 Hz (Figure 4A, right inset). Moreover, Pr5 and Sp5C neurons were identified as wide dynamic range (WDR) neurons according to their response to tactile and nociceptive (noxious heating of their orofacial RF) stimulation. Nociceptive-specific Sp5C neurons were not included in this study.

All trigeminal neurons included in this study displayed an ipsilateral RF that corresponded to one-two whiskers for Pr5 neurons or two-five whiskers for Sp5C neurons. Tactile responses to deflection of one whisker in Pr5 neurons consisted of 1–3 spikes per stimulus with a mean response of 1.4 ± 0.17 spikes/stimulus. However, Sp5C neurons displayed a larger RF with a mean response of 1.9 ± 0.25 spikes/stimulus (Figures 4B, 4C; left histograms). Response latency was slightly shorter in Pr5 neurons than in Sp5C neurons (7.6 ± 0.27 ms and 9.2 ± 0.12 ms, respectively).

Electrical stimulation of contralateral S1 cortex with a single stimulus (10–100 µA; 0.3 ms duration) induced orthodromic discharges of 1–2 spikes at latencies of 4.9 ± 0.5 ms in Pr5 neurons (*n* = 52) or 5.7 ± 0.4 ms in Sp5C neurons (*n* = 38; Figures 4B, 4C; right histograms). S2 cortical stimulation (30–100 µA; 0.3 ms duration) also induced orthodromic discharges of 1–2 spikes at latencies of 5.2 ± 0.4 ms (*n* = 12) and 5.8 ± 0.6 ms (*n* = 19) in Pr5 and in Sp5C nuclei, respectively.

### S1 or S2 cortical effects on Pr5 neurons

**Figure 5:**
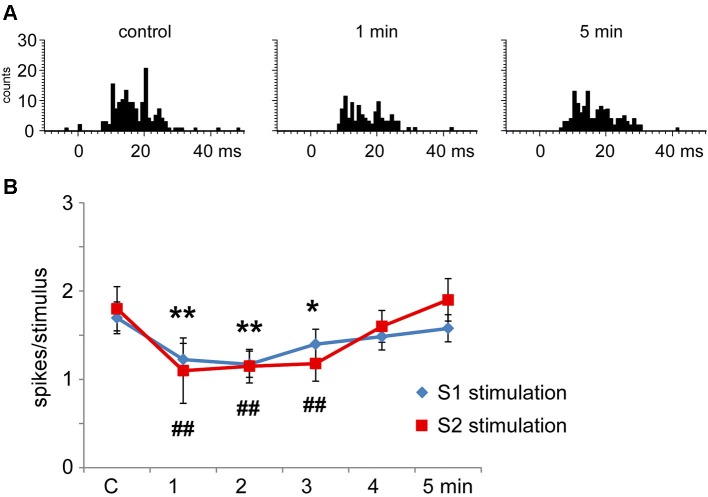
**Long-lasting S1 and S2 cortical effects on Pr5 neurons in unmatching conditions. (A)** histograms show PSTHs in control condition, 1 min and 5 min after a stimulation train in a representative Pr5 neuron. **(B)** plot of the mean tactile responses in control condition and after S1 (blue diamonds) or S2 (red squares) cortical electrical stimulation (3 trains of stimuli; 50 Hz during 500 ms repeated every 2 s) in Pr5 neurons recorded in an unmatched condition (*n* = 35 and *n* = 9, respectively). Cortical stimulation inhibited tactile responses for 3 min. In this and in the following figures * *P* < 0.05, ** *P* < 0.01 statistical significance after S1 stimulation train with respect to control values; # *P* < 0.05, ## *P* < 0.01 after S2 stimulation train.

S1 cortical stimulation (3 trains of stimuli at 50 Hz during 500 ms repeated each 2 s) induced long-lasting changes in tactile responses according to the stimulated cortical RF. When cortical and Pr5 RFs were different (unmatched condition) most of the Pr5 neurons (35 out of 45 neurons; 78%) showed a statistically significant decrease in tactile responses after S1 stimulation trains (from 1.7 ± 0.3 spikes/stimulus in control conditions to 1.2 ± 0.2 spikes/stimulus; *n* = 40, *p* = 0.002, 1 min after cortical trains; Figure 5A). The effect lasted at least 3 min and tactile responses recovered control levels later on (1.6 ± 0.1 spikes/stimulus 5 min after cortical trains; Figure 5B). The cortical stimulation did not induce a long-lasting firing inhibition, probably because of the low firing rate in spontaneous conditions. The remaining neurons (10 out of 45 neurons; 22%) did not change the tactile response after the S1 stimulation.

Multiunit recordings in S2 cortex revealed larger RFs in the whisker pad than in S1 cortex, so the RFs received inputs from several whiskers. Electrical stimulation of contralateral S2 cortex (3 trains of stimuli at 50 Hz during 500 ms repeated each 2 s) in the unmatched condition induced long-lasting inhibition of tactile responses in 69% of Pr5 neurons (9 out of 13 neurons; Figure 5B); the remaining 4 neurons were not affected (31%). Figure 5B shows that control tactile responses decreased from 1.8 ± 0.2 spikes/stimulus in control conditions to 1.1 ± 0.4 spikes/stimulus (*n* = 9; *p* = 0.006) at 1 min, returning to control levels 5 min after cortical stimulation (1.6 ± 0.3 spikes/stimulus).

**Figure 6:**
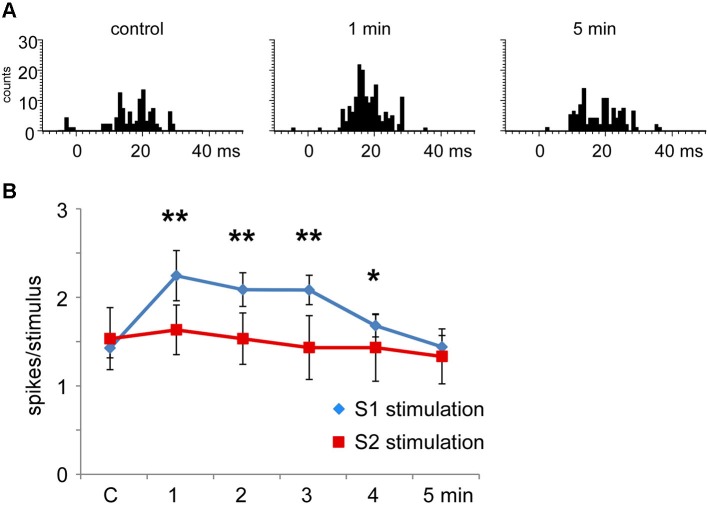
**Long-lasting S1 and S2 cortical effects on Pr5 neurons in matching conditions. (A)** histograms show PSTHs in control condition, 1 min and 5 min after stimulation train in a representative Pr5 neuron. **(B)** plot of the mean tactile responses in control condition and after S1 (blue diamonds) or S2 (red squares) cortical electrical stimulation (3 trains of stimuli; 50 Hz during 500 ms repeated each 2 s) in Pr5 neurons recorded in the matched condition. Pr5 neurons increased their tactile response after a stimulation train in the S1 cortex (*n* = 21). A similar stimulation train in the S2 cortex did not modify tactile responses in Pr5 neurons (*n* = 12). * *P* < 0.05, ** *P* < 0.01 statistical significance after cortical stimulation train with respect to control values.

When the RFs of the stimulated cortical area and the Pr5 cells overlapped (matched condition) most Pr5 neurons increased their tactile response (21 out of 30 neurons; 70%; Figure 6A), but a third of them were unaffected (9 out of 30 neurons; 30%). When the tactile response was facilitated the mean tactile response increased from 1.5 ± 0.1 spikes/stimulus in control conditions to 2.3 ± 0.3 spikes/stimulus (*n* = 21; *p* = 0.001) 1 min after the cortical stimulating train, and control levels were recovered 5 min later (1.4 ± 0.3 spikes/stimulus; Figure 6B). In the matched condition, S2 electrical stimulation induced a tactile facilitation in a single Pr5 neuron (8%) while most of the neurons were not affected (*n* = 12; 92%; Figure 6B).

### Pharmacological studies on Pr5 neurons

**Figure 7:**
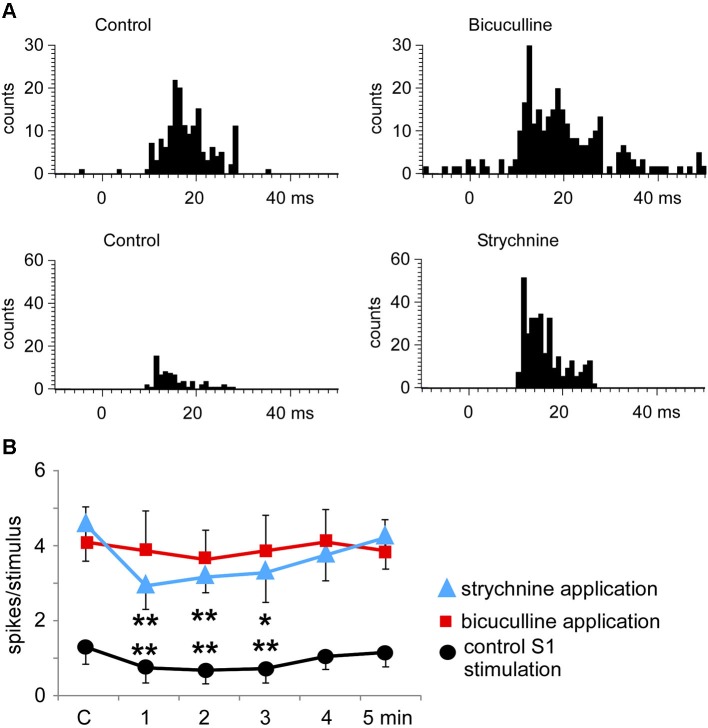
**Bicuculline and strychnine increase tactile responses of Pr5 neurons. (A)** PSTHs of a representative neuron in control conditions and after iontophoretic application of bicuculline in the Pr5 nucleus (10 mM; 50 nA; upper histograms). Bicuculline blocked GABAergic inhibition and the tactile responses increased. A similar effect was observed after application of the glycinergic receptor antagonist strychnine (100 mM; 100 nA; lower histograms). **(B)** plot of the mean tactile responses before (C) and 1–5 min after the application of an SI stimulation train. SI cortical stimulation inhibited Pr5 tactile responses after application of saline solution (*n* = 12; black circles), but the effect was blocked by bicuculline (*n* = 9; red squares). However, strychnine application did not affect the long-lasting cortical-evoked inhibition (*n* = 7; blue triangles). * *P* < 0.05, ** *P* < 0.01 statistical significance after cortical stimulation train with respect to control values.

The above results indicated that S1 as well as S2 stimulation induced a long-lasting inhibition of tactile responses in most Pr5 neurons in unmatched conditions. To determine the mechanism of this inhibition the GABA_A_ receptor antagonist bicuculline was iontophoretically applied into the Pr5 nucleus (10 mM; 50 nA). Bicuculline strongly increased the spontaneous firing rate of Pr5 neurons (from 0.4 ± 0.2 spikes/s in controls to 6.3 ± 1.4 spikes/s in 9 Pr5 neurons, *p* < 0.001) and tactile responses (from 1.7 ± 0.2 to 4.9 ± 1.2 spikes/stimulus, *p* < 0.001; Figure 7A). Moreover, the inhibition in tactile responses evoked by either S1 or S2 cortical stimulation was abolished in the presence of bicuculline, indicating that GABA_A_ receptors were involved in the generation of the corticofugal-evoked inhibition (Figure 7B, red symbols).

Application of the glycinergic receptor antagonist strychnine (100 mM; 100 nA) also increased the spontaneous firing rate (from 0.5 ± 0.2 spikes/s in control to 3.1 ± 1.6 spikes/s, *p* < 0.001) and tactile responses in 7 Pr5 neurons (from 1.7 ± 0.2 to 4.5 ± 0.7 spikes/stimulus, *p* < 0.001; Figure 7A). However, the inhibition evoked by S1 or S2 cortical stimulation was not modified (Figure 7B, blue symbols), indicating that glycinergic receptors were not responsible for the long-lasting effect of the corticofugal inhibition in Pr5 nucleus.

**Figure 8:**
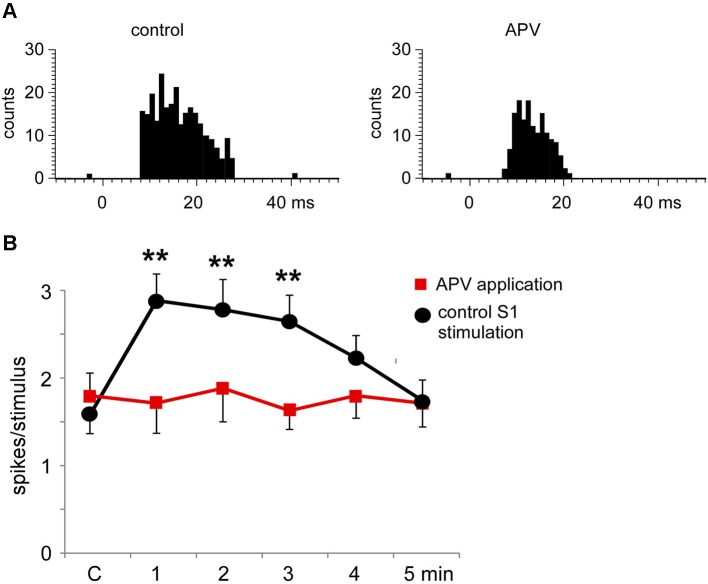
**The long-lasting facilitation evoked by S1 cortical stimulation was due to NMDA receptor activation. (A)** PSTHs of tactile responses in control conditions and after iontophoretic application of APV (50 mM, 100 nA) in the Pr5 nucleus. In the presence of APV, tactile responses were reduced. **(B)** Plot of the mean tactile responses before (C) and 1–5 min after the application of a SI stimulation train in a matched condition. In the presence of APV (*n* = 14 cells; red squares) the long-lasting facilitation evoked by corticofugal stimulation was blocked in comparison to responses after the application of saline solution (*n* = 8 cells; black circles). ** *P* < 0.01 statistical significance after cortical stimulation train with respect to control values.

The S1 cortical stimulation induced facilitation of tactile responses in Pr5 neurons in the matched condition, as shown above. Iontophoretic application of the NMDA receptor antagonist APV (50 mM, 100 nA) in the Pr5 nucleus reduced tactile responses in Pr5 neurons (*n* = 14; Figure 8A) and blocked the long-lasting facilitation evoked by corticofugal stimulation (Figure 8B).

### Nociceptive stimulation induced changes in the activity of Pr5 and Sp5C neurons

**Figure 9:**
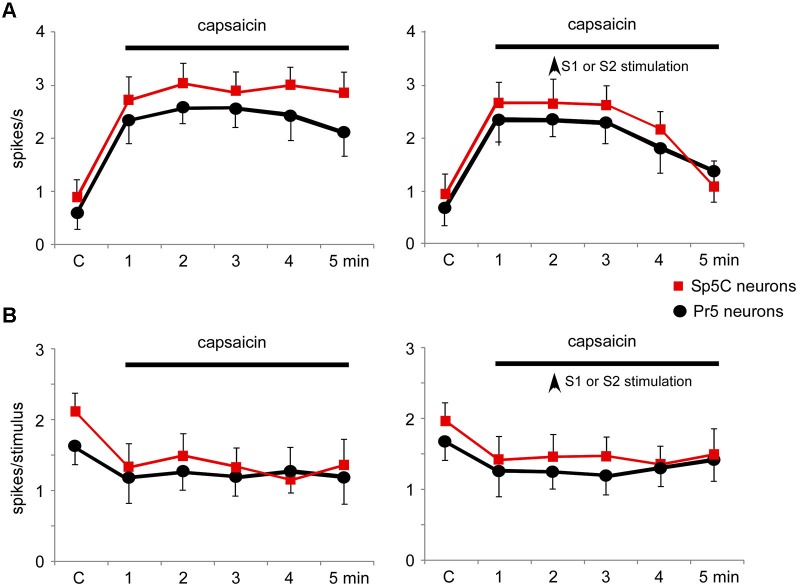
**Nociceptive stimulation modified the firing pattern of Pr5 and Sp5C neurons and was partially reverted by S1 or S2 cortical stimulation. (A)** Plot of the firing rate of Pr5 (black circles) and Sp5C (red squares) after application of capsaicin cream on the whisker pad. The firing rate increased in both types of neurons (left plot). Electrical stimulation (3 trains of stimuli; 50 Hz during 500 ms repeated each 2 s) of S1 or S2 cortex (vertical arrow) inhibited the capsaicin-evoked increase of the firing rate (right plot). **(B)** Plot of the tactile responses of Pr5 (black circles) and Sp5C (red squares) after application of capsaicin cream on the whisker pad. Tactile responses decreased in both types of neurons. Electrical stimulation (3 trains of stimuli; 50 Hz during 500 ms repeated every 2 s) of S1 or S2 cortex (vertical arrow) did not modify the capsaicin-evoked decrease in tactile responses.

Topical application of capsaicin cream induced a long-lasting increase of the spontaneous firing rate in the Pr5 (from 0.5 ± 0.28 spikes/s to 2.1 ± 0.9 spikes/s; %; *p* = 0.001; *n* = 14) and Sp5C neurons (from 0.9 ± 0.32 spikes/s to 3.8 ± 0.64 spikes/s; 283%; *p* < 0.001; *n* = 22) 5 min after capsaicin application (Figure 9A, left plot). The effect remained as long as the time the capsaicin cream was on the whisker pad. Tactile stimuli deflecting the whisker at the RF (20 ms duration) evoked 1.6 ± 0.23 spikes/stimulus in Pr5 (*n* = 14 neurons) or 2.2 ± 0.19 spikes/stimulus in Sp5C (*n* = 22) neurons in control conditions. Tactile responses decreased 5 min after capsaicin application to 1.2 ± 0.15 spikes/stimulus (*p* = 0.03) or 1.3 ± 0.18 spikes/stimulus (*p* = 0.007), respectively (Figure 9B, left plot).

The increases in the trigeminal firing rate induced by capsaicin were reduced significantly by either S1 (*n* = 8) or S2 (*n* = 4) cortical stimulation (Figure 9A, right plot). Three minutes after cortical stimulation neuronal activity recovered control values. In contrast, the reduction in tactile responses evoked by capsaicin was not affected by cortical stimulation (Figure 9B, right plot).

## Discussion

The main finding of the present study is that somatosensory cortex exerts a control over the somatosensory responses to non-nociceptive and nociceptive stimuli in the Pr5 and Sp5C nuclei. The somatosensory cortex may modulate tactile responses in the Pr5 nucleus by excitatory and inhibitory actions depending on the neuronal RF. Cortical neurons mediate both the excitatory feedback to subcortical neurons with overlapping RFs and a widespread inhibition to “unmatched neurons” (see for review Nuñez and Malmierca, 2007). This cortical feedback can play a relevant role in control the sensory information reaching the thalamus and cortex. In contrast, the somatosensory cortex only inhibits nociceptive responses in the Pr5 and Sp5C nucleus (see also Malmierca et al., 2012).

Trigeminal neurons showed a slow spontaneous activity that was correlated with the slow EEG activity recorded simultaneously. This slow oscillation was described by Steriade et al. (1993) and has been recorded in numerous subcortical structures related with the cortex such as the basal forebrain (Nuñez, 1996) or the cuneatus neurons (Mariño et al., 2000), indicating that corticofugal projections may modulate their activity.

Direct projections from the whisker barrel areas in the S1 to all levels of the trigeminal complex have been demonstrated by means of anterograde tract-tracing methods in mice (Wise and Jones, 1977; Welker et al., 1988). Earlier studies labeling primary afferents innervating the trigeminal complex have revealed that the somatotopic arrangement of orofacial projections from S1 cortex to the trigeminal nucleus is very similar to that of central projections of primary afferents arising from the trigeminal ganglion neurons that project mainly to the ipsilateral trigeminal complex (Hayashi, 1985; Shigenaga et al., 1989; Arvidsson and Rice, 1991; Takemura et al., 1991; Tomita et al., 2012). Present results further demonstrate that the cingulate, somatosensory and Ins cortices have projections that exclusively target the Pr5 nucleus, probably to control the transmission of non-nociceptive tactile stimuli, as has been suggested by the electrophysiological results. Moreover, cortical neurons projecting to the Sp5C nucleus also project to the Pr5 nucleus, suggesting there is not an exclusive corticofugal pathway from the cingulate, somatosensory or Ins cortices to the Sp5C nuclei that only modulate nociceptive responses. Somatotopic organization of tactile and nociceptive RFs has been described in the S1 (Lamour et al., 1983a, b; Kenshalo et al., 2000) and operculo-insular cortices (Brooks et al., 2005; Mazzola et al., 2009; Baumgartner et al., 2010), which project to the Sp5C nuclei. However, these studies only observe a rough somatotopy of the nociceptive responses. Thus, the inhibitory influence of the cortex on nociceptive responses in the trigeminal complex would arise from somatosensory and insular cortices, but would be organized in a non-discriminatory manner, in contrast to the precise organization of coticofugal projections to Pr5.

Previous results have indicated that inputs from nociceptive or WDR thalamic neurons to rat S1 cortex are not segregated into anatomically distinct cortical regions (Monconduit et al., 2006). In contrast, in monkeys nociceptive S1 neurons are somatotopically organized with the majority of nociceptive neurons located in the middle layers (III and IV) of area 1 (Kenshalo et al., 2000). Intermixed populations of non-nociceptive and nociceptive neurons might be linked by intrinsic local connections within S1, thus allowing interactions between somatosensory submodalities. It is interesting to note that Cg cortex that is involved in nociception does not project to Sp5C nucleus, probably because this cortex is involved in emotional aspects of pain and not in the location and intensity of the nociceptive stimulus (Petrovic et al., 2004).

Tactile responses were facilitated by somatosensory cortical stimulation when the activated cortical area and trigeminal neuron RFs overlapped (“matched” RF). In contrast, tactile responses were inhibited when the cortical and trigeminal RFs were non-overlapping. Similar effects have been described in the dorsal column nuclei cells (Malmierca and Nuñez, 1998, 2004; Canedo and Aguilar, 2000; Aguilar et al., 2003), suggesting that S1 cortex may enhance relevant tactile stimuli while simultaneously inhibiting irrelevant stimuli from the limbs (in dorsal column nuclei) or from orofacial areas, as has been described here (see for review Nuñez and Malmierca, 2007). Electrical stimulation of the S1 cortex evokes facilitation or inhibition of Pr5 neurons according to the RF of the cortical area stimulated. This is supported by earlier electrophysiological studies indicating that the somatosensory cortex provides feedback projections to the Pr5 and Sp5C in the cat (Dubner and Sessle, 1971) as well as to the trigeminal nucleus in rats (Woolston et al., 1983; Furuta et al., 2010; Noseda et al., 2010). The terminals of the corticofugal projections may be glutamate-immunopositive (Donoghue et al., 1985; Giuffrida and Rustioni, 1989; Valtschanoff et al., 1993), indicating that they are excitatory. Thus, inhibitory influences may be evoked by local interneurons. Topographically, the corticofugal S1 projection is characterized by an efferent distribution arising from a single barrel to reach the Pr5 nucleus (Welker et al., 1988). Accordingly, we observed groups of labeled neurons in layer V of the barrel zone that projected to the trigeminal nuclei while the septa contained only few cells that projected to the trigeminal complex.

The long-lasting facilitatory effect of S1 on Pr5 neurons was due to activation of NMDA receptors since it was blocked by application of the NMDA receptor antagonist APV while inhibition was due to activation of GABA_A_ receptors because it was blocked by the GABA_A_ receptor antagonist bicuculline. It has been reported previously, and was corroborated in this study, that S1 inhibits nociceptive responses in Sp5C neurons by activation of glycinergic and GABAergic receptors (Malmierca et al., 2012). Strychnine and bicuculline increased the spontaneous activity, as also shown in the gracilis and cuneatus nuclei (Aguilar et al., 2003; Malmierca and Nuñez, 2004; Leiras et al., 2010), indicating that glycine and GABA tonically inhibit neuronal activity probably filtering weak, irrelevant synaptic inputs. However, strychnine did not affect the corticofugal-evoked long-lasting inhibition of Pr5 neurons. The participation of glycinergic neurons in the control of sensory transmission in the somatosensory pathway is complicated. Aguilar et al. (2003) demonstrated that GABAergic neurons were controlled by glycinergic neurons within the cuneatus nucleus. When the glycinergic neurons were activated by corticofugal projections they inhibited GABAergic neurons and consequently enhancing tactile responses. The same glycinergic control may occur in the Pr5 nucleus because tactile responses increased after strychnine application within Pr5 nucleus, while the inhibition evoked by S1 or S2 cortical stimulation was not affected.

Glycinergic or GABAergic inhibitory neurotransmission also plays a pivotal role in the modulation of pain signals in Sp5C neurons (Malmierca et al., 2012; for review see Furue et al., 2004). In contrast to the action in the Pr5 nucleus, the S1 or S2 cortical effect on Sp5C neurons was only inhibition, as was suggested previously (Malmierca et al., 2012), probably because nociceptive stimuli are always behaviorally relevant and the cortex is not interested in “tuning” that somatosensory information. However, Noseda et al. (2010) demonstrated that S1 cortex may induce facilitation or inhibition of meningeal-evoked responses, indicating that cortical modulation of nociceptive responses may be vary according to the sensory information being transmitted. Taking into account that corticofugal projections reaches the Pr5 and Sp5C nuclei and that inhibitory neurons have been described in both nuclei, we suggest that the origin of the whisker response inhibition here described may be due to the activity of local neurons within the trigeminal complex. Further studies would be necessary to clarify this hypothesis.

To best of our knowledge the participation of glycinergic transmission in the control of tactile responses in Pr5 nucleus had not yet been reported although glycinergic neurons and terminals are densely observed in all of the trigeminal nuclei including the Pr5 nucleus (Rampon et al., 1996). Our results indicate that glycinergic synaptic transmission is not involved in the corticofugal-evoked inhibition of non-nociceptive responses on Pr5 neurons. However, glycinergic synaptic transmission participates in the corticofugal-evoked inhibition of nociceptive responses on Sp5C neurons. Thus, our findings reveal that S1 and S2 are able to selectively modulate different somatosensory submodalities (nociceptive or non-nociceptive stimuli) through specific anatomical projections on local interneurons and specific actions on trigeminal nuclei (excitatory and inhibitory influences on Pr5 nucleus or only inhibitory influences on Sp5C nucleus). A similar result was published by Leiras et al. (2010). Proprioceptive responses could be inhibited through GABAergic and glycinergic interneurons in the midventral cuneate nucleus, while tactile responses were inhibited by GABAergic synaptic inputs and enhanced by glycinergic synaptic inputs (Aguilar et al., 2003). Therefore, somatosensory cortex could contribute to the detection of innocuous and nociceptive cutaneous inputs through specific tuning mechanisms acting simultaneously in the Pr5 and Sp5C nuclei.

A previous study has demonstrated that the orofacial S2 area projects somatotopically to regions in the trigeminal complex (Haque et al., 2012). The orofacial area of S2 cortex projects contralaterally not only to the Pr5 or the oral subnucleus of the trigeminal sensory nuclear complex but also to some levels of the trigeminal interpolar subnucleus and the Sp5C, with a somatotopic pattern in a dorsoventral direction and in a superficial–deep direction within all trigeminal levels. Present data indicate that electrical stimulation of S2 cortex inhibits both Pr5 and Sp5C neurons independently of whether the cortical and trigeminal RFs overlapped. It is possible that the lack of Pr5 nucleus facilitation observed after S2 stimulation when cortical and trigeminal RFs overlapped might be due to the S2 cortical RFs being more diffuse than in S1 cortex. This would make it difficult to stimulate the exact cortical spot that projects to specific trigeminal neurons with overlapping RFs. Also, it is possible that S2 corticofugal projections lack the necessary fine wiring to modulate specific subcortical neurons as occur in the S1 cortex or in the primary visual or auditory cortex (e.g., Cudeiro and Sillito, 2006; Antunes et al., 2010; Anderson and Malmierca, 2013).

As in the auditory and visual systems, corticofugal influences from somatosensory cortex serve to amplify the effects of sensory stimulation to the classic center-surrounding RFs and help to sharpen and adjust the profile of RFs (“egocentric selection”) (Rauschecker, 1995; Suga and Ma, 2003; Nuñez and Malmierca, 2007). Consequently, the anatomical pattern of these connections is complicate (for review see Feldmeyer, 2012). Different cortical layers are implicated in the processing specific aspects of sensory information. Thus, corticofugal projections from these layers have different targets in subcortical structures. Particularly, cortical neurons innervating trigeminal nuclei are located in the layer Vb, which also innervates the ipsilateral motor and S2 cortices, the contralateral S1 cortex as well as the posteromedial thalamic nucleus. Taken together, previous and present results indicate that somatosensory corticofugal neurons can contribute to sensory processing by selecting trigeminal responses as preferred or non-preferred inputs, in a modality-specific manner, and so contribute to the perception of specifically sensory sensations.

An interesting point is that nociceptive modulation could be performed at different levels of the somatosensory pathway. Recent results suggest that nociceptive responses in the Sp5C may be modulated by supraspinal mechanisms such as serotoninergic projections to Sp5C (Okubo et al., 2013) or from the cortex (Malmierca et al., 2012; present Results section). Motor cortex project to different motor nuclei of the thalamus (Urbain and Deschênes, 2007; Alloway et al., 2008). This projection may be involved in somatosensory response modulation during exploratory whisking (Urbain and Deschênes, 2007). Also, somatosensory and motor cortices project to the posterior medial nucleus of the thalamus in rodents, in which convey nociceptive and non-nociceptive inputs (Veinante et al., 2000; Alloway et al., 2008; Wu et al., 2013). Moreover, motor or prefrontal cortical stimulation is a relatively recent neurosurgical technique for pain control (Kenshalo and Willis, 1991; Masri and Keller, 2012; Wager et al., 2013). At least 50% of patients with chronic, pharmaco-resistant neuropathic pain may benefit from this technique. However, the mechanisms of action remain elusive (Hardy, 1985; Garcia-Larrea and Peyron, 2007). Our results did not show corticofugal projections from motor cortex to the Sp5C nucleus, suggesting that the reduction of pain by motor stimulation could be due to activation of cortico-cortical projections to somatosensory cortices, which clearly project to brainstem centers involved in pain sensory processing. However, the analgesic effects observed in animals and humans by motor cortex stimulation may be also due to corticofugal projections from the motor cortex to the sensory thalamic nuclei or zona incerta (Lucas et al., 2011; Masri and Keller, 2012). Taken together all data suggest that somatosensory cortex may modulate sensory responses at the brainstem level while motor cortex may influence sensory responses at thalamic level.

## Author contributions

Eduardo Malmierca, Irene Chaves-Coira, Margarita Rodrigo-Angulo and Angel Nuñez conception and design of research; Eduardo Malmierca and Irene Chaves-Coira performed experiments and analyzed data; Eduardo Malmierca, Irene Chaves-Coira, Margarita Rodrigo-Angulo and Angel Nuñez interpreted results of experiments and drafted manuscript; Eduardo Malmierca, Irene Chaves-Coira, Margarita Rodrigo-Angulo and Angel Nuñez approved final version of manuscript.

## Conflict of interest statement

The authors declare that the research was conducted in the absence of any commercial or financial relationships that could be construed as a potential conflict of interest.
